# Binding of Two Intrinsically Disordered Peptides to a Multi-Specific Protein: A Combined Monte Carlo and Molecular Dynamics Study

**DOI:** 10.1371/journal.pcbi.1002682

**Published:** 2012-09-13

**Authors:** Iskra Staneva, Yongqi Huang, Zhirong Liu, Stefan Wallin

**Affiliations:** 1 Department of Astronomy and Theoretical Physics, Computational Biology and Biological Physics group, Lund University, Lund, Sweden; 2 College of Chemistry and Molecular Engineering, and Center for Quantitative Biology, Peking University, Beijing, China; University of California San Diego, United States of America

## Abstract

The unique ability of intrinsically disordered proteins (IDPs) to fold upon binding to partner molecules makes them functionally well-suited for cellular communication networks. For example, the folding-binding of different IDP sequences onto the same surface of an ordered protein provides a mechanism for signaling in a many-to-one manner. Here, we study the molecular details of this signaling mechanism by applying both Molecular Dynamics and Monte Carlo methods to S100B, a calcium-modulated homodimeric protein, and two of its IDP targets, p53 and TRTK-12. Despite adopting somewhat different conformations in complex with S100B and showing no apparent sequence similarity, the two IDP targets associate in virtually the same manner. As free chains, both target sequences remain flexible and sample their respective bound, natively 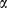-helical states to a small extent. Association occurs through an intermediate state in the periphery of the S100B binding pocket, stabilized by nonnative interactions which are either hydrophobic or electrostatic in nature. Our results highlight the importance of overall physical properties of IDP segments, such as net charge or presence of strongly hydrophobic amino acids, for molecular recognition via coupled folding-binding.

## Author Summary

A substantial fraction of our proteins are believed to be partly or completely disordered, meaning that they contain regions that lack a stable folded structure under typical physiological conditions. This is a feature which plays a key role in their functions. For example, it allows them to have many structurally different binding partners which in turn permits the construction of the intricate signaling and regulatory networks necessary to sustain complex biological organisms such as ourselves. Whereas measuring the binding strengths of associations involving disordered proteins is routine, the binding process itself is today still not fully understood. We use two different computational models to study the interactions of a folded protein, S100B, which can bind various disordered peptides. In particular, we compare two peptides whose structures are known when in complex with S100B. Our results suggest that, although the peptides assume different structures in the bound state, there are similarities in how they associate with S100B. The possibility to computationally model the interplay between proteins is an important complement to experiments, by identifying crucial steps in the binding process. This is essential to understand, e.g., how single mutations sometimes lead to serious diseases.

## Introduction

It has become clear that many functional proteins do not fold into unique three-dimensional structures, as expected from the classical view of proteins, but remain highly conformationally dynamic under native conditions. These intrinsically disordered proteins (IDPs) are now estimated to represent a significant fraction of many genomes. For example, roughly half of the proteins in mammals are predicted to contain disordered segments of more than 30 amino acids in length, and a fourth to be fully disordered [1]. Interestingly, the prevalence of disorder is far from uniform among different functional classes, suggesting IDPs carry special and biologically relevant properties. In particular, the prevalence is high among proteins performing important regulatory and signaling functions [2].

The perhaps most striking IDP property is their ability to undergo a conformational disorder-order transition upon contact with a target molecule. Various biological advantages have been suggested to stem directly from this coupled folding-binding process [3]–[6], including the ability to bind specifically to multiple and structurally diverse partners [7]. Indeed, intrinsic disorder has been found to be a common feature of “hub” proteins with especially large numbers of links in protein interaction networks [8], [9]. An important example is given by the tumor suppressor p53, a transcription factor and regulatory protein that can induce cell cycle arrest and apoptosis [10]. The C terminal domain of p53 (henceforth simply p53) binds to at least four different globular proteins adopting four different conformations in the process [11]–[13]. How this binding diversity is achieved at the molecular level is not well understood and, in particular, cannot be rationalized by the classical lock-and-key model of protein-protein interactions.

The binding diversity of IDPs can in principle lead to two different types of signaling modes, one-to-many, in which one disordered region binds to multiple folded proteins, and many-to-one, in which many disordered segments bind to the same folded protein [14]. An example of the latter is given by S100B, a 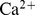-modulated homodimeric protein with several disordered protein partners [15]–[17], one of which is p53. Each S100B monomer consists of two EF-hand motifs which upon 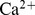-binding reorients one 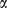-helix (helix III), thereby exposing a hydrophobic binding pocket. In order to better understand this multiple specificity phenomenon, we study the coupled folding-binding of two disordered peptide sequences to S100B. As target peptides we choose p53 and a fragment from the protein CapZ, denoted TRTK-12, which both have available experimental structural data for the complexes [16], [18]–[20]. Molecular Dynamics (MD) simulation studies have previously been performed on the disordered N and C terminal regions of p53, with either positional constraints on the peptide [21] or by focusing on the characteristics of the bound and free states separately [22]–[24]. There has, however, not been a study comparing the processes of different IDP segments binding to the same ordered target.

MD simulations of biomolecular systems are typically hampered by limitations in conformational sampling. Large dynamical transitions such as coupled folding-binding of proteins is particularly challenging. We circumvent the problem by using a computationally efficient Monte Carlo-based implicit-solvent model, which has been extensively tested on the folding of peptides and small proteins [25]–[27]. Our basic approach has previously been tested on PDZ domain-peptide binding, with a variation of the potential energy function [28], [29]. One advantage of this approach over recent protein-peptide docking methods [30]–[32] is that an equilibrium picture of the association is obtained. Additionally, we perform explicit-water MD simulations on IDP segments as free chains and in complex with S100B to further validate the MC result. Our work suggests striking similarities in the association mechanism of p53 and TRTK-12, despite their different amino acid sequences. In particular, we find that both sequences populate a transient intermediate state which is stabilized by either hydrophobic or electrostatic interactions.

## Results/Discussion

Using an all-atom MC procedure, described in detail in Methods, we perform extensive fixed-temperature simulations to characterize the interaction between the 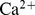-loaded form of the S100B homodimer and the two disordered peptides p53 (positions 374–388 of the full-length p53) and TRTK-12 (positions 265–276 of CapZ). In one-letter code, the amino acid sequences are GQSTSRHKKLMFKTE and TRTKIDWNKILS for p53 and TRTK-12, respectively. These peptides were chosen because they are well-characterized targets of S100B, and the interaction with p53 has previously been studied computationally [22], [23]. The S100B structural forms used in the MC simulations are taken from the respective p53 and TRTK-12 bound structures but no specific knowledge of the binding pocket is built in. Free energy surfaces of the folding-binding processes are constructed and used to define bound and intermediate states which are then characterized in detail. Additional explicit-water MD simulations are also performed on free peptides, as well as on selected bound structures, to test the validity of our approach.

### Conformational Preferences of Free Peptides

**Figure 1:**
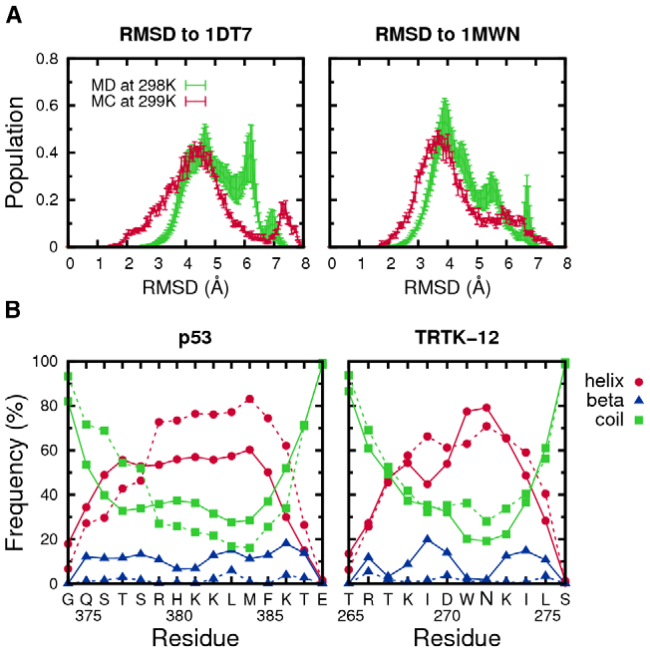
Conformational preferences of the free p53 and TKTK-12 peptide sequences. (A) Distributions of RMSD for p53 and TRTK-12, as obtained by both implicit-water MC and explicit-water MD simulations, where RMSD is calculated with regard to the experimentally determined bound structures for the respective peptides (PDB IDs 1DT7 and 1MWN). (B) Secondary structure content profiles for p53 and TRTK-12, analyzed using STRIDE [56], presented separately for the MC (solid lines) and MD (dashed line) simulations. The seven different secondary structure categories have been grouped into three main classes: helix (STRIDE notations h, g, i, t), 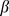-sheet (e, b) and coil (c).

We start by examining the conformational behavior of our two peptide sequences as free chains using both MC and MD simulations, respectively (see Methods). Despite no discernable sequence similarity between the p53 and TRTK-12 peptides, we find that the two chains behave qualitatively similarly, as seen from Figure 1. Both peptides remain rather flexible at temperatures 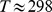 K and sample a wide range of conformations. A slight preference for 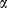-helical states can be seen. Consequently, the conformational fluctuations of the free peptide chains include structures resembling the bound state. These bound-like populations are nonetheless quite low (see the low value tails of the RMSD distributions), in agreement with previous MD simulations on the p53 peptide [21].

We also find that the results of the two different simulation approaches are in good qualitative agreement. This is important as a validation of the full-scale protein-peptide simulations to be described below. The largest deviation appears for the p53 sequence, for which the MD results produce a peak in the RMSD probability distribution at around 6 Å. By inspection, we see that this peak represents peptide chains folded into a 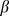-hairpin-like structure. The MC simulations of p53 also produce a small population of 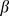-hairpins, albeit typically more fully formed, resulting in a shift of the peak to around 7.5 Å (see Figure 1A). We note that the tendency for the free p53 peptide to form 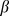-hairpin-like structures was also observed in the MD study of Allen et al. [22]. Overall, we find that both the free p53 and TRTK-12 peptides sample multiple conformations, including transient 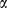-helical structures.

### Binding Free Energy Surfaces

We turn now to the binding processes of S100B and the p53 and TRTK-12 peptides, respectively. To this end, we rely on the computational convenience of the MC approach. The basic procedure follows earlier work [28], [29] and operates in the following way. The S100B dimer structure is maintained in its native state by constraints, allowing side-chain and small backbone motions, while the peptide chains are free to explore the protein surface. Simulations are performed at fixed temperatures and are long enough to produce multiple binding and unbinding events in each trajectory such that an equilibrium picture of the interaction is obtained. Representative MC trajectories are shown in Figure S1 in Supporting Information.

To monitor the progress of binding we use two different observables, 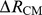 and 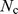. The distance 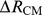 is taken between two points, the center-of-mass of the simulated peptide and the center-of-mass obtained from the peptide coordinates in the experimental complex structure. Hence, a small 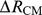 value indicates binding close to the experimentally determined binding site. The other observable, 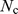, is the number of amino acid contacts between the peptide chain and the S100B peptide binding pocket, which we have defined as a set of amino acid surface positions (see Methods). Because of the symmetry of the S100B homodimer, with its two identical binding pockets, we determine the center-of-mass distance using
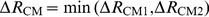
(1)where 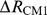 and 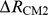 are obtained for the two binding pockets, respectively. For 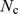, we count the total contacts made with either binding pocket residues, thus assuming that a peptide can not contact both sites at the same time. This way, high 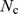 and low 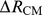 is a signature of a peptide bound tightly at either of the two binding pockets.

**Figure 2:**
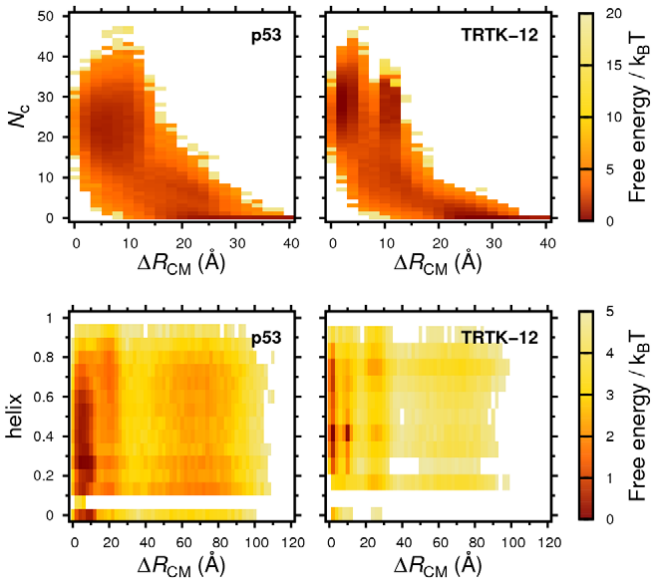
Folding and binding free energy landscapes of p53 and TRTK-12. The progress of binding is measured by two observables, 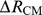 and 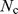, (see text) and folding by the fraction of helical content (defined as in Figure 1). Free energies are obtained from extensive fixed-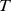 equilibrium MC simulations of the interaction between peptide and S100B, in which several reversible binding and unbinding events occur, where 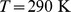 and 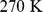 for for p53 and TRTK-12, respectively.

The overall features of the obtained folding and binding processes can be seen from the free energy surfaces in Figure 2. Both sequences exhibit major free energy minima representing bound states, characterized by 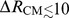 Å and 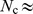
20–40. This indicates that binding occurs mainly at the experimentally determined S100B peptide binding pockets. We emphasize that this result is obtained in unbiased simulations, in which the peptides are left free to explore the entire protein surface.

**Figure 3:**
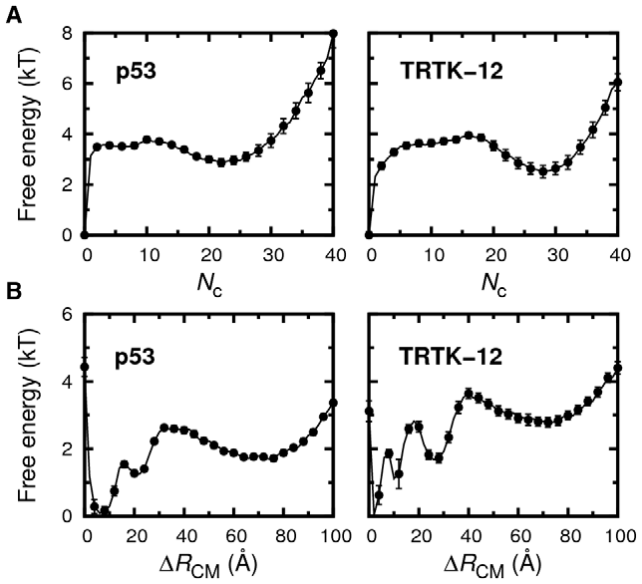
The binding of p53 and TRTK-12 to S100B. Free energy profiles of binding observables (A) 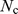 and (B) 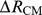, obtained from fixed-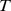 MC simulations. Statistical errors are calculated using the jackknife method [58].

We find additional, more detailed similarities between the binding processes of the p53 and TRTK-12 sequences. In the bound state, both p53 and TRTK-12 exhibit fluctuating helical contents. Hence, the peptides display a significant population of bound but not yet folded structures. This population is apparent in the one-dimensional free energy profiles in Figure 3, which reveal a plateau-like behavior in 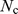 representing an intermediate state between the fully bound and free states. The term “encounter complex” has been used to indicate such a metastable state in protein-protein interactions [33]. For coupled folding-binding of disordered chains, there are two extreme mechanistic possibilities for association corresponding to either folding-before-binding (conformational selection) or binding-before-folding (induced folding) of the peptide [34]. The significant population of “non-native” bound peptide conformations we observe for p53 and TRTK-12 indicate a binding mechanism dominated by induced-folding for both sequences. Similar observations have been made in the studies of Chen [21] and Pirolli et al. [23] for the p53 sequence, as well as for associations involving other IDPs [35]–[37].

In previous studies on PDZ domain-peptide interactions we found a two-state-like binding mechanism [28], [29], i.e., where the bound state was reached via a single well-defined binding transition state. The difference with the current systems may be a length effect, as PDZ domains typically bind much shorter peptides (around 4–6 amino acids). These short chains may not have sufficient chain flexibility to promote the formation of intermediate states [38].

### The Bound State Ensemble: Structure and Protein-Peptide Contacts

**Figure 4:**
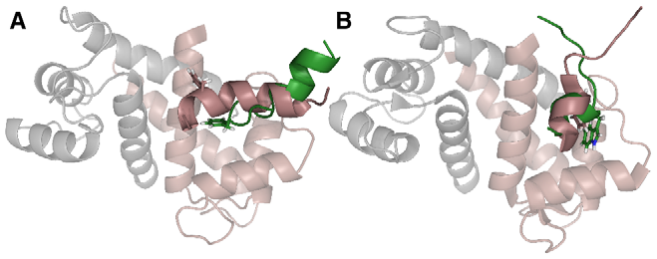
Minimum-energy structures obtained from unbiased MC simulations of the interaction between S100B and its targets. The minimum-energy conformations, across all MC trajectories, obtained for (A) p53 and (B) TRTK-12 are shown in green. Experimental structures of the p53-S100B (1D7T) [16] and TRTK-12-S100B (1MWN) [19] complexes are displayed in gray and pink. The “non-optimized” root-mean-square deviation between the minimum-energy and experimental peptide structures are 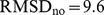 and 7.3 Å for p53 and TRTK-12, respectively (see Methods). The figure was prepared with PyMOL [59].

X-ray and NMR structural studies on p53 [16] and TRTK-12 [19], [20] show that both peptides bind the hydrophobic surface on S100B, exposed upon 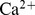-binding, but with somewhat different configurations. In complex with S100B, the p53 peptide is almost fully helical and docked parallel with the S100B helix III while TRTK-12 has only a single 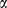-helical turn and is oriented perpendicular to helix III. The two minimum-energy conformations obtained from our MC binding simulations of p53 and TRTK-12, respectively, manage to capture many of these characteristics as shown in Figure 4. The TRTK-12 peptide in particular has in effect assumed the correct structure, being only partly helical and its tryptophan (Trp271) sidechain directed into the hydrophobic binding pocket. The min-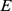 conformation of p53 deviates slightly from the experimental structure. As depicted in Figure 4A, its phenylalanine (Phe385) is contacting the hydrophobic pocket rather than being solvent exposed, as in the experimental structure [16]. This hinders the p53 peptide from being fully 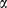-helical. However, the overall chain orientation relative to helix III is essentially correct.

It is clear from the binding free energy surfaces in Figure 2 that both peptides exhibit a significant structural diversity in the bound state. Minimum-energy structures may therefore not always provide a complete picture. The p53 peptide has a single, smooth bound state free-energy minimum spanning 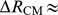
2–10 Å. It turns out that this state is structurally quite well represented by the p53 min-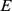 conformation in Figure 4A, with contacts between the S100B binding pocket and the p53 Phe385 as a prominent feature. By contrast, TRTK-12 has two distinct bound state minima characterized by 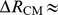
2–5 Å and 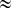
10 Å, respectively. The underlying structures of the small-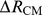 (global) free energy minimum are similar to the min-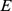 conformation. Interestingly, the local minimum at 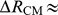
10 Å exhibited by TRTK-12 corresponds to an 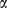-helix parallel to the S100B helix III, reminiscent of the experimental binding pose of p53 [16].

**Figure 5:**
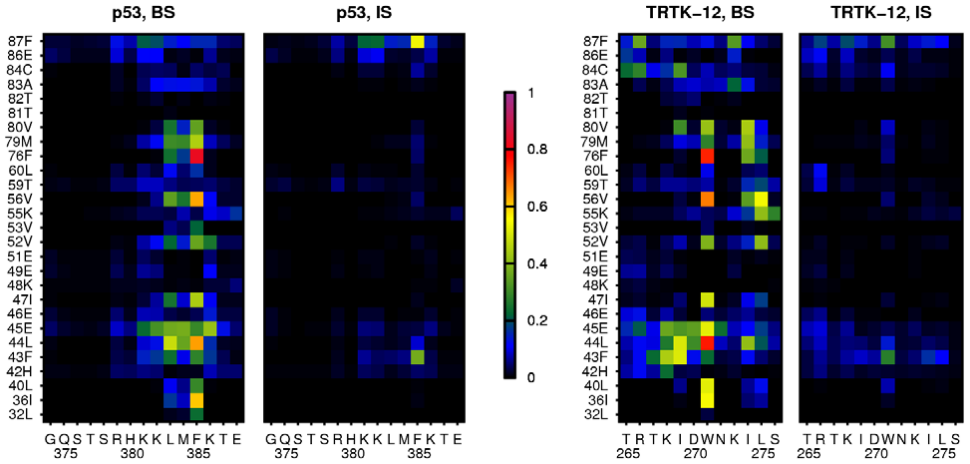
Probabilistic contact maps for the interaction between S100B and its peptide targets taken separately for the bound (BS) and intermediate (IS) states. The color scale indicate the probability of contact formation between peptide amino acids (horizontal axes) and amino acids of the S100B peptide binding pocket (vertical axes).

**Figure 6:**
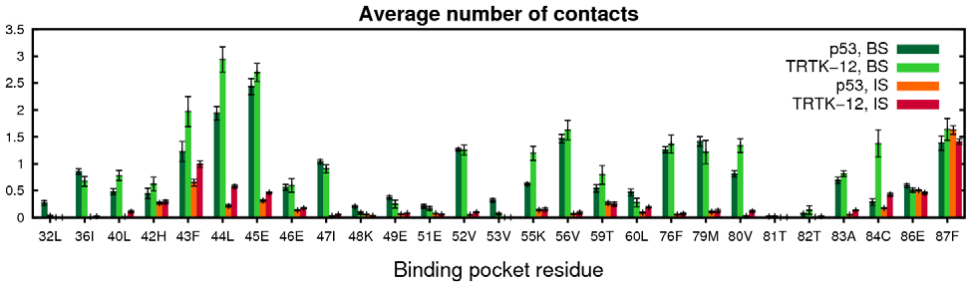
Involvement in binding for different amino acid positions of the S100B binding pocket. For each S100B position, the number of contacted amino acids on the peptide is counted. Shown are the average numbers obtained separately for the bound (BS) and intermediate (IS) states. Statistical errors are estimated using the jackknife method [58].

To make a more detailed comparison with experimental data, we define the bound state (BS) by 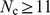 and 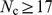 for the p53 and TRTK-12 systems, respectively, based on the free energy profiles in Figure 3A. The populations of all possible amino acid contacts in the BS between the peptide and the S100B binding pocket are illustrated in Figure 5. p53 mainly binds to the binding pocket via its hydrophobic residues Leu383, Met384 and Phe385, in contrast to TRTK-12 which interacts with all its amino acids. This difference aside, the simulations suggest that the largest hydrophobic residue in each peptide fulfills a similar function. For p53, Phe385 is involved in all of the most populated contacts, equivalent to Trp271 in TRTK-12. These residues interact mainly with the hydrophobic amino acids of the binding pocket, especially with Ile36, Leu44, Val56 and Phe76. Our simulations suggest more involvement of the p53 Phe385 than what at first glance is apparent from the NMR structure. However, the importance of this position for binding is implied in the study of Rustandi et al. [39], who found that mutation of the Phe to a Trp, a larger hydrophobic amino acid, increases the binding affinity by 3–4 fold. As demonstrated in Figure 6, most S100B binding-pocket residues make on average the same number of contacts with p53 as with TRTK-12. There are however some positions, in particular Lys55 and Cys84, which are mostly involved in binding TRTK-12. These two residues indeed form contacts with TRTK-12 in the experimental structures [19], [20], which we further find to be stable also in MD simulations of the S100B-TRTK-12 complex, performed as described below (see Figure S2).

While there is an overlap between the obtained contact maps in Figure 5 and the set of interactions derived from experiments, our BS ensembles do not perfectly fit with the experimental NMR structures. Some residue-residue contacts are only present in the simulations and, conversely, some contacts are found in the experimental structures but not in the simulations. The latter mainly emerges in the p53 simulations, where the two inter-molecular salt bridges (Arg379–Glu45 and Lys386–Glu86), as well as contacts involving the N-terminal part of the p53 chain, are mostly absent in our simulations. The reason is probably the rather frequent interaction of Phe385 with the hydrophobic S100B binding pocket, which lead to a disrupted folding of the p53 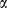-helix. Our BS conformational ensembles are also more diverse than the set of NMR derived model structures. This can be seen by calculating the average RMSD between pairs of structures ij in the NMR and BS ensembles, respectively. For p53, we obtain 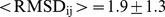 Å for the NMR ensemble of S100B-p53 structures and 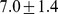 Å for the simulation-derived BS ensemble. The corresponding values for TRTK-12 are 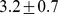 Å and 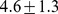 Å, respectively. It is possible therefore that our model is not able to capture the folding of the peptides in full, at least for p53. There is some uncertainty, however, in the interpretation of the nuclear Overhauser enhancements (NOEs) intensities which underly NMR structure calculations, especially with regard to the diversity of the protein ensemble [40], . The differences may also be due to the relatively weak binding conditions used in our simulations, where the BS is populated only at around 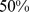. At lower temperatures, the BS ensemble will likely become structurally more well-defined.

### Testing Proposed Structures of the S100B-TRTK-12 Complex

In the analysis of our binding simulations we rely on available experimental structures of the protein-peptide complexes. For S100B and TRTK-12, three structures have been proposed [18]–[20]. One of these involves a novel coil-like conformation of TRTK-12 (PDB ID 1MQ1) [18] while the NMR and X-ray structures of Weber *et al.* both indicate a partially 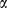-helical conformation (PDB ID 1MWN and 3IQQ) [19], [20]. Our MC results are in line with the presence of a short 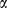-helix in TRTK-12, as demonstrated by the minimum-energy conformation in Figure 4B.

**Figure 7:**
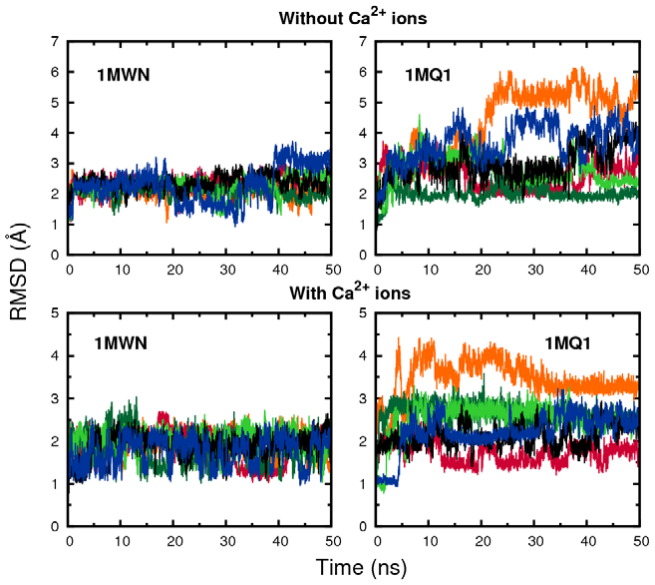
Stabilities of two alternative native state structures for the S100B–TRTK-12 complex. Results are shown for four sets of MD simulations, obtained by starting from two different NMR structures of the complex, 1MWN or 1MQ1, and performed either with or without 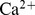 present. In case of absent 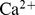 ions, additional constraints on the S100B dimer are included (see Methods). RMSD values are calculated for the structured part of the TRTK-12 peptide, positions 5–12.

To further inspect the experimental disagreement in TRTK-12 structure, we employed our MD approach to test the stability of two of the proposed structures, 1MQ1 [18] and 1MWN [19]. Three simulations of 50 ns were performed for each complex, both with and without 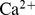 loaded in S100B, as shown in Figure 7. The calculations consistently indicate a higher stability for 1MWN than for 1MQ1, at least in terms of RMSD fluctuation measured over the TRTK-12 chain. The peptide secondary structure in the 1MWN trajectories remains intact over the full MD trajectories (data not shown). These MD trajectories thus also support the partly 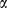-helical structural models of Weber *et al.* [19], [20] and, moreover, provide further support for an agreement between our two different computational approaches.

### Characterizing the Intermediate State

How do the peptide chains reach their respective bound states or, in other words, what is the peptide binding mechanism? Key to answering this question is partly bound peptide conformations populated transiently during the binding process. Although we cannot directly assess kinetic aspects of the binding process with our MC approach, we can examine the structural characteristics of peptide conformations which are neither entirely bound nor in the unbound state. As shown above, both sequences exhibit similar plateau-like regions in the 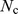 free energy profiles (see Figure 3). Many of these conformations must be populated during binding and it is therefore of interest to characterize and compare these states. We define this intermediate state, IS, as conformations which are neither unbound (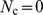) nor part of the bound state, BS.

**Figure 8:**
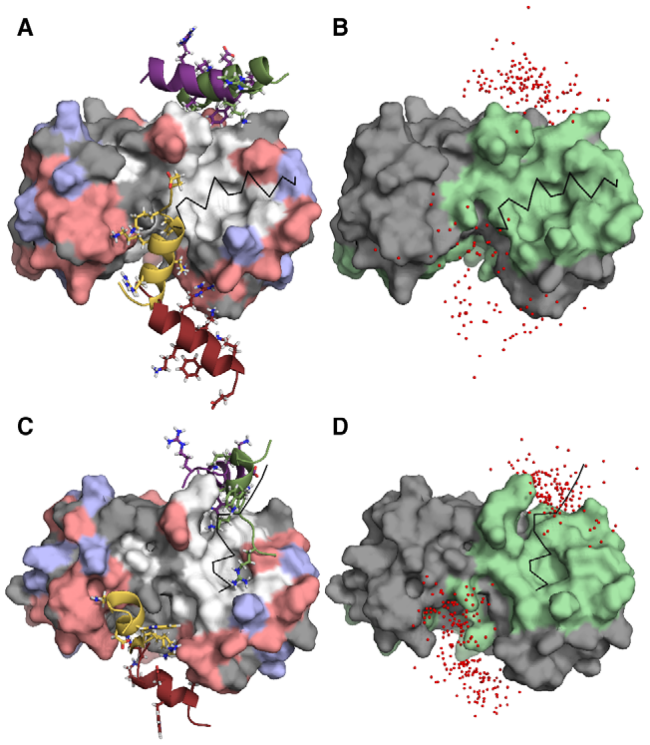
Illustration of the p53 and TRTK-12 intermediate states, IS. A structural cluster procedure is performed on the IS ensembles, as discussed in the text. Representative peptide structures (cluster centroids) of the four largest clusters are displayed for (A) p53 and (C) TRTK-12. The S100B homodimer is displayed as a molecular surface where positively charged amino acids (Arg, Lys) are shown in blue, negatively charged (Asp, Glu) in red, and hydrophobic (Met, Leu, Ala, Ile, Val, Phe) in white. The experimental bound peptide structures are outlined as thin black backbone traces. The center-of-mass points of all peptide structures in the four clusters are also shown for (B) p53 and (D) TRTK-12, where the amino acids of the S100B binding pocket are colored in green. The figure was prepared with PyMOL [59].

Contrary to the BS ensembles, the IS of the two peptide sequences exhibit strong similarities. To show this, we performed a clustering procedure on all IS conformations with the aim of visualizing typical structures (see Methods for details). The four largest clusters for p53 and TRTK-12, respectively, are displayed in Figure 8, representing more than half of the IS ensemble in both cases. As depicted in Figures 8A and C, the IS peptide conformations are located primarily in two different regions in the periphery of the S100B binding pocket. In the first group, represented by the violet and the green cluster centroid structures in Figure 8, the peptides are located in the vicinity of a hydrophobic surface composed primarily of Phe43 and Phe87 on the S100B domain. Contacts between these amino acids and the peptides are also evident from the IS contact probabilities in Figure 5, which moreover indicate that they involve mainly the peptide residues Phe385 and Trp271 on p53 and TRTK-12, respectively. Interestingly, while these contacts are among the most frequent in IS, they are much less populated in the BS. In the second IS group, indicated by the red and the yellow centroids in Figure 8, the peptides are situated between helix III and helix IV of S100B. Here the stabilizing interactions are instead primarily electrostatic. The possible existence of binding spots in the periphery of the binding pocket is in part supported by the work of Arendt *et al.* [42], who found that small molecules from a fragment library mainly bound to two sites of the S100B surface, one of which overlaps with the first region described here.

The similar binding mechanism of p53 and TRTK-12 is especially interesting in light of their low sequence similarity and the significant structural differences in their bound states. The S100B interaction with disordered peptides can be compared with so-called peptide recognition domains (PRDs), which are common modules in signaling proteins and include 14-3-3, PDZ, and SH3 [43]. These domains typically bind sets of peptide sequences conforming to simple linear sequence motifs. For example, SH3 domains bind peptides comprising a Pro-X-X-Pro motif, where Pro is proline and X any amino acid type. A structural analysis of the many-to-one binding exhibited by a particular 14-3-3 domain revealed a relatively high structural similarity in the protein-peptide complexes involving five different target peptides [14]. Similarly, PDZ domains typically bind their peptides in a structurally specific way involving the peptide C terminus [44]. The multiple specificity exhibited by S100B thus differ from PRDs in at least two ways. First, the peptide sequences do not conform to a simple linear motif and, second, their bound structures differ significantly. A possible biological benefit of these two properties is that cross-reactivity among different S100B target peptides may be minimized. In other words, the relatively high sequence disparity among the IDP targets keep them from acquiring each others general functions. For example, the difference in sequence between CapZ/TRTK-12 and p53 may keep CapZ/TRTK-12 from binding to p53 partner domains other than S100B.

Despite the lack of a simple sequence motif for known S100B targets, we note some interesting common characteristics. Both p53 and TRTK-12 include a single aromatic amino acid (Phe385 and Trp271, respectively), one or more aliphatic amino acids, as well as several Lys and Arg resulting in a net positive charge of the peptide. These characteristics holds also for a third disordered S100B target for which a structure of the complex is available [45], taken from the N terminal regulatory domain of NDR kinase. It is possible that this basic compositional similarity of target peptides underpins the similarity in the binding mechanism, such that interactions made early in the binding process, being diverse and nonspecific in nature, are largely determined by the overall physical properties of the chain segment. An issue which remains to be addressed is to what extent a common binding mechanism is a general characteristics of many-to-one protein signaling. For this comprehensive additional simulations beyond the scope of the present investigation are required. However, we note that preliminary simulations for the interaction between S100B and NDR, performed using our all-atom MC approach, produced a minimum-energy conformation where the (sole) aromatic residue of the NDR peptide is involved in binding in a similar way as for p53 and TRTK-12 (see Figure S3).

### Summary and Conclusion

We have used a combination of MD and MC all-atom simulations to understand the coupled folding and binding of two disordered peptides, p53 and TRTK-12, to the 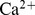-loaded form of S100B, as an example of a many-to-one signaling mechanism. Despite the significant difference in sequence, we find remarkable and unexpected similarities in their association behavior with S100B. First, as free peptides, the two sequences have similar predispositions towards helical conformations, but overall remain rather flexible chains. Only to a small extent do they sample their respective S100B-bound structures. Second, both p53 and TRTK-12 populate an intermediate, metastable state during the folding-binding process, which may serve as an initial encounter complex of the interaction. The intermediate state is divided structurally between two different binding surfaces on the periphery of the binding pocket where stabilization occur by either hydrophobic or electrostatic interactions and includes a significant fraction of 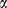-helical structure (see Figure 8). Interestingly, major disparities between the two S100B targets become apparent only in the final bound state, where the patterns of contacts with the S100B surface are significantly dissimilar.

## Methods

### Molecular Dynamics Simulations

MD simulations of the free p53 and TRTK-12 peptides were started from random conformations which were obtained from high temperature (400 K) MD simulations. 20 simulations were performed for p53 and TRTK-12, respectively. The duration for each simulation was 200 ns.

The simulations of the 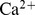
–S100B–TRTK-12 complex were initialized from the two available NMR structures (PDB IDs 1MWN and 1MQ1). For each version three runs of 50 ns, with different initial velocity distributions, were performed. In 1MQ1, the coordinates for the four 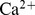 ions are missing. They were therefore added by superimposing the structure of 1MWN on 1MQ1 and transferring the coordinates of the 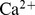 ions from 1MWN to 1MQ1. A final manual adjustment of the ion positions was made in order to avoid steric repulsions with the proteins. We also performed three simulations of each complex variant without the 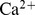 ions present. In order to maintain the structure of S100B, position restraints on the backbone atoms were added.

All simulations were performed using the program GROMACS 4.07 [46], [47] with the OPLS-AA/L force field [48] and the SPC/E water model [49]. The starting conformation was placed in the center of a cubic water box with at least 10 Å from the box edge. Periodic boundary conditions were used and counter ions (Na^+^ or Cl^−^) were added to neutralize the net charges. The long-range electrostatic interactions were treated with the particle mesh Ewald method [50]. The cutoff distances were set to 10 Å for short-range Coulomb and van der Waals interactions. The bond lengths were fixed by the LINCS algorithm [51], and a time step of 2 fs was used. Each system was first relaxed by 1000 steps of steepest-descent energy minimization. After the minimization, the system was equilibrated at 298 K for 100 ps under an NVT ensemble and further equilibrated for 200 ps at constant pressure (1 bar). V-rescale [52] and Parrinello-Rahman [53] were used to couple the system to the simulation temperature and pressure with coupling constants of 0.1 ps and 2.0 ps, respectively. Production simulations were performed at constant temperature (298 K) and pressure (1 bar). Coordinates were saved every 5 ps.

### Monte Carlo Simulations

For the free peptides, 10 simulations were performed with the PROFASI program package [25]–[27] using a simulated tempering algorithm [54], [55]. Each run consisted of 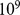 MC steps and visited 6 temperatures in the range 280–330 K for p53 and 201–301 K for TRTK-12. The temperature intervals were chosen to envelop the protein–peptide simulation temperatures.

10 simulations were also performed for each S100B-peptide system, where every run consisted of 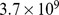 MC steps. All binding simulations were performed in a cube with side length 150 Å and periodic boundary conditions. The temperature 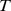 was held fixed, chosen such that the peptide would be bound roughly half of the time (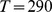 K for p53 and 270 K for TRTK-12). The protein had flexible backbone and sidechains, but was kept close to its native bound conformation (taken from the NMR structures with PDB IDs 1DT7 and 1MWN, where the best representative conformers were chosen). This was done by using a total energy function 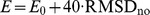, where 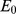 is the original physical energy function defined in Ref. [27]. Sampling of protein conformations close to the S100B native state was ensured by the non-optimized root-mean-square deviation penalty term given by
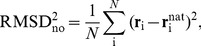
(2)where 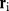 and 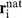 are the positions of the C*_α_* atom of amino acid i in the simulated and experimental protein structures, respectively, and 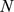 is the number of amino acids in the protein. The additional energy term constitutes typically less than 25% of the total energy in the system. Four flexible N-terminal residues, Phe88–Glu91, of each S100B monomer were removed.

### Peptide Folding

The average amount of secondary structure in the free peptides was determined using STRIDE assignments [56]. The similarity to the natively bound structure was analyzed by calculations of the peptide backbone RMSD (translationally and rotationally optimized) with respect to the best representative conformer of the NMR structures (1DT7 for p53 and 1MWN for TRTK-12).

### Binding Observables

As an indication of whether the peptide is bound to the correct binding site, we used the observables 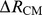 and 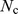. 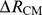 is the distance between the centers-of-mass of the simulated and the experimental peptide in the complex, respectively. 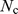 is the number of contacts between the peptide and the protein binding pocket, where two residues are defined to be in contact if they exhibit at least two pairs of heavy atoms separated by less than 4.5 Å. The residues defining the protein binding pocket are listed in Figure 5.

### Clustering Procedure

In order to characterize the most populated structural states of the intermediate, we used a Complete Linkage Hierarchical clustering method [57]. The aim of this procedure is to group protein-peptide conformations that are similar to each other into clusters. To determine similarity we used the standard root-mean-square-deviation measure, RMSD. The cutoff value in the clustering procedure was taken to be 6 Å and 8 Å for TRTK-12 and p53, respectively, which resulted in roughly the same number of clusters for both sequences. The clustering procedure guarantees that within each cluster, the RMSD between all pairs of conformations is less than the chosen cutoff value.

## Supporting Information

Figure S1
**Representative MC trajectories of the binding and unbinding of S100B and its peptide targets.** Shown is the MC evolution of 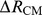 for two of the total 20 independent simulations performed for (A) p53 and (B) TRTK-12.(EPS)

Figure S2 **Time evolution of residue-residue distances in MD trajectories of the S100B-TRTK-12 complex.** Distances are calculated between the sidechain center-of-mass points of the involved residues, Lys55 and Cys84 for S100B and Ile5 and Leu11 for TRTK-12.
(EPS)

Figure S3
**MC protein-peptide binding simulation of S100B and the N terminal regulatory domain of NDR kinase.** (A) MC evolution of 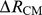 (see Equation 1 in main text) showing two independent binding/unbinding events, and (B) the minimum-energy conformation found during the trajectory where the simulated peptide is shown in green and the experimental protein-peptide structure is shown in gray and pink (PDB ID 1PSB) [45]. The NDR peptide sequence used in the simulation is KETEFLRLKRTRLGLE.(EPS)

## References

[1] Dunker A, Silman I, Uversky V, Sussman J (2008) Function and structure of inherently disordered proteins. Curr Opin Struc Biol 18: 756–764. 10.1016/j.sbi.2008.10.002 18952168

[2] Iakoucheva LM, Brown CJ, Lawson JD, Obradović Z, Dunker AK (2002) Intrinsic disorder in cell-signaling and cancer-associated proteins. J Mol Biol 323: 573–584. 12381310 10.1016/s0022-2836(02)00969-5

[3] Dunker AK, Garner E, Guilliot S, Romero P, Albrecht K, et al (1998) Protein disorder and the evolution of molecular recognition: theory, predictions and observations. Pac Symp Biocomput 1998: 473–484. 9697205

[4] Shoemaker BA, Portman JJ, Wolynes PG (2000) Speeding molecular recognition by using the folding funnel: the y-casting mechanism. Proc Natl Acad Sci US A 97: 8868–8873. 10.1073/pnas.160259697 PMC16787 10908673

[5] Gunasekaran K, Tsai CJ, Kumar S, Zanuy D, Nussinov R (2003) Extended disordered proteins: targeting function with less scaffold. Trends Biochem Sci 28: 81–85. 12575995 10.1016/S0968-0004(03)00003-3

[6] Hilser VJ, Thompson EB (2007) Intrinsic disorder as a mechanism to optimize allosteric coupling in proteins. Proc Natl Acad Sci U S A 104: 8311–8315. 17494761 10.1073/pnas.0700329104 PMC1895946

[7] Kriwacki RW, Hengst L, Tennant L, Reed SI, Wright PE (1996) Structural studies of p21Waf1/Cip1/Sdi1 in the free and Cdk2-bound state: conformational disorder mediates binding diversity. Proc Natl Acad Sci U S A 93: 11504–11509. 8876165 10.1073/pnas.93.21.11504 PMC38087

[8] Ekman D, Light S, Björklund ÅK, Elofsson A (2006) What properties characterize the hub proteins of the protein-protein interaction network of Saccharomyces cerevisiae? Genome Biol 7: R45. 16780599 10.1186/gb-2006-7-6-r45 PMC1779539

[9] Haynes C, Oldfield CJ, Ji F, Klitgord N, Cusick ME, et al (2006) Intrinsic disorder is a common feature of hub proteins from four eukaryotic interactomes. PLoS Comput Biol 2: e100. 16884331 10.1371/journal.pcbi.0020100 PMC1526461

[10] Bálint È, Vousden KH (2001) Activation and activities of the p53 tumour suppressor protein. Brit J Cancer 85: 1813–1823. 11747320 10.1054/bjoc.2001.2128 PMC2364002

[11] Avalos JL, Celic I, Muhammad S, Cosgrove MS, Boeke JD, et al (2002) Structure of a Sir 2 enzyme bound to an acetylated p53 peptide. Mol Cell 10: 523–535. 12408821 10.1016/s1097-2765(02)00628-7

[12] Lowe ED, Tews I, Cheng KY, Brown NR, Gul S, et al (2002) Specificity determinants of recruitment peptides bound to phospho-CDK2/cyclin A. Biochemistry 41: 15625–15634. 12501191 10.1021/bi0268910

[13] Mujtaba S, He Y, Zeng L, Yan S, Plotnikova O, et al (2004) Structural mechanism of the bromodomain of the coactivator CBP in p53 transcriptional activation. Mol Cell 13: 251–263. 14759370 10.1016/s1097-2765(03)00528-8

[14] Oldfield CJ, Meng J, Yang JY, Yang MQ, Uversky VN, et al (2008) Flexible nets: disorder and induced fit in the associations of p53 and 14-3-3 with their partners. BMC Genomics 9 Suppl 1: S1. 10.1186/1471-2164-9-S1-S1 PMC2386051 18366598

[15] Dempsey B, Shaw G (2011) Identification of calcium-dependent and calcium-enhanced binding between S100B and the dopamine D2 receptor. Biochemistry 50: 9056–9065. 21932834 10.1021/bi201054x PMC3196243

[16] Rustandi RR, Baldisseri DM, Weber DJ (2000) Structure of the negative regulatory domain of p53 bound to S100B(*β β*). Nature Struct Biol 7: 570–574. 10876243 10.1038/76797

[17] Barber KR, McClintock KA, Jamieson GA Jr, Dimlich RVW, Shaw GS (1999) Specificity and Zn^2+^ enhancement of the S100B binding epitope TRTK-12. J Biol Chem 274: 1502–1508. 9880526 10.1074/jbc.274.3.1502

[18] McClintock K, Shaw G (2003) A novel target conformation is revealed by the solution structure of the Ca^2+^–S100B–TRTK-12 complex. J Biol Chem 278: 6251–6257. 12480931 10.1074/jbc.M210622200

[19] Inman K, Yang R, Rustandi R, Miller K, Baldisseri D, et al (2002) Solution NMR structure of S100B bound to the high-affinity target peptide TRTK-12. J Mol Biol 324: 1003–1014. 12470955 10.1016/s0022-2836(02)01152-x

[20] Charpentier T, Thompson L, Liriano M, Varney KM, Wilder P, et al (2010) The effects of CapZ peptide (TRTK-12) binding to S100B-Ca^2+^ as examined by NMR and X-ray crystallography. J Mol Biol 396: 1227–1243. 20053360 10.1016/j.jmb.2009.12.057 PMC2843395

[21] Chen J (2009) Intrinsically disordered p53 extreme C-terminus binds to S100B(*β β*) through “fly-casting”. J Am Chem Soc 131: 2088–2089. 19216110 10.1021/ja809547p

[22] Allen W, Capelluto D, Finkelstein C, Bevan D (2010) Modeling the relationship between the p53 C-terminal domain and its binding partners using molecular dynamics. J Phys Chem 114: 13201–13213. 10.1021/jp1011445 20873738

[23] Pirolli D, Alinovi CC, Capoluongo E, Satta MA, Concolino P, et al (2011) Insight into a novel p53 single point mutation (G389E) by Molecular Dynamics simulations. Int J Mol Sci 12: 128–140. 10.3390/ijms12010128 PMC3039947 21339981

[24] Huang Y, Liu Z (2011) Anchoring Intrinsically Disordered Proteins to Multiple Targets: Lessons from N-Terminus of the p53 Protein. Int J Mol Sci 12: 1410–1430. 21541066 10.3390/ijms12021410 PMC3083713

[25] Irbäck A, Samuelsson B, Sjunnesson F, Wallin S (2003) Thermodynamics of alpha- and betastructure formation in proteins. Biophys J 85: 1466–1473. 12944264 10.1016/S0006-3495(03)74579-2 PMC1303323

[26] Irbäck A, Mohanty S (2006) PROFASI: A Monte Carlo simulation package for protein folding and aggregation. J Comput Chem 27: 1548–1555. 16847934 10.1002/jcc.20452

[27] Irbäck A, Mitternacht S, Mohanty S (2009) An effective all-atom potential for proteins. PMC Biophys 2: 2. 19356242 10.1186/1757-5036-2-2 PMC2696411

[28] Staneva I, Wallin S (2009) All-atom Monte Carlo approach to protein-peptide binding. J Mol Biol 393: 1118–1128. 19733177 10.1016/j.jmb.2009.08.063

[29] Staneva I, Wallin S (2011) Binding free energy landscape of domain-peptide interactions. PLoS Comput Biol 7: e1002131. 21876662 10.1371/journal.pcbi.1002131 PMC3158039

[30] Petsalaki E, Stark A, García-Urdiales E, Russell RB (2009) Accurate prediction of peptide binding sites on protein surfaces. PLoS Comput Biol 5: e1000335. 19325869 10.1371/journal.pcbi.1000335 PMC2653190

[31] Raveh B, London N, Zimmerman L, Schueler-Furman O (2011) Rosetta FlexPepDock ab-initio: simultaneous folding, docking and refinement of peptides onto their receptors. PLoS ONE 6: e18934. 21572516 10.1371/journal.pone.0018934 PMC3084719

[32] Dagliyan O, Proctor EA, D'Auria KM, Ding F, Dokholyan NV (2011) Structural and dynamic determinants of protein-peptide recognition. Structure 19: 1837–1845. 22153506 10.1016/j.str.2011.09.014 PMC3240807

[33] Schreiber G (2002) Kinetic studies of protein-protein interactions. Curr Opin Struct Biol 12: 41–47. 11839488 10.1016/s0959-440x(02)00287-7

[34] Wright PE, Dyson HJ (2009) Linking folding and binding. Curr Opin Struct Biol 19: 31–38. 19157855 10.1016/j.sbi.2008.12.003 PMC2675572

[35] Sugase K, Dyson HJ, Wright PE (2007) Mechanism of coupled folding and binding of an intrinsically disordered protein. Nature 447: 1021–1025. 17522630 10.1038/nature05858

[36] Ahmad M, Gu W, Helms V (2008) Mechanism of fast peptide recognition by SH3 domains. Angew Chem Int Ed Engl 47: 7626–7630. 18752238 10.1002/anie.200801856

[37] Huang Y, Liu Z (2009) Kinetic Advantage of Intrinsically Disordered Proteins in Coupled FoldingBinding Process: A Critical Assessment of the “Fly-Casting” Mechanism. J Mol Biol 393: 1143–1159. 19747922 10.1016/j.jmb.2009.09.010

[38] Huang Y, Liu Z (2010) Nonnative interactions in coupled folding and binding processes of intrinsically disordered proteins. PLoS ONE 5: e15375. 21079758 10.1371/journal.pone.0015375 PMC2973977

[39] Rustandi RR, Drohat AC, Baldisseri DM, Wilder PT, Weber DJ (1998) The Ca^2+^-dependent interaction of S100(*β β*) with a peptide derived from p53. Biochemistry 37: 1951–1960. 9485322 10.1021/bi972701n

[40] Burgi R, Pitera J, van Gunsteren WF (2001) Assessing the effect of conformational averaging on the measured values of observables. J Biomol NMR 19: 305–320. 11370777 10.1023/a:1011295422203

[41] Zagrovic B, van Gunsteren WF (2006) Comparing atomistic simulation data with the NMR experiment: how much can NOEs actually tell us? Proteins 63: 210–218. 16425239 10.1002/prot.20872

[42] Arendt Y, Bhaumik A, Del Conte R, Luchinat C, Mori M, et al (2007) Fragment docking to S100 proteins reveals a wide diversity of weak interaction sites. Chem Med Chem 2: 1648–1654. 17705319 10.1002/cmdc.200700096

[43] Pawson T, Nash P (2003) Assembly of cell regulatory systems through protein interaction domains. Science 300: 445–452. 12702867 10.1126/science.1083653

[44] Lee HJ, Zheng JJ (2010) PDZ domains and their binding partners: structure, specificity, and modification. Cell Commun Signal 8: 8. 20509869 10.1186/1478-811X-8-8 PMC2891790

[45] Bhattacharya S, Large E, Heizmann CW, Hemmings B, Chazin WJ (2003) Structure of the Ca^2+^/S100B/NDR kinase peptide complex: insights into S100 target specificity and activation of the kinase. Biochemistry 42: 14416–14426. 14661952 10.1021/bi035089a

[46] Berendsen H, van der Spoel D, Drunen R (1995) GROMACS: a message-passing parallel molecular dynamics implementation. Comp Phys Comm 91: 43–56.

[47] Hess B, Kutzner C, van der Spoel D, Lindahl E (2008) GROMACS 4: algorithms for highly efficient, load-balanced, and scalable molecular simulations. J Chem Theory Comput 4: 435–447. 26620784 10.1021/ct700301q

[48] Kaminski G, Friesner R, Tirado-Rives J, Jorgensen W (2001) Evaluation and reparametrization of the OPLS-AA force field for proteins via comparison with accurate quantum chemical calculations on peptides. J Phys Chem B 105: 6474–6487.

[49] Berendsen H, Grigera J, Straatsma T (1987) The missing term in effective pair potentials. J Phys Chem 91: 6269–6271.

[50] Darden T, York D, Pedersen L (1993) Particle mesh Ewald: an N⋅log(N) method for Ewald sums in large systems. J Chem Phys 98: 10089–10092.

[51] Hess B, Bekker H, Berendsen H, Fraaije J (1997) LINCS: a linear constraint solver for molecular simulations. J Comput Chem 18: 1463–1472.

[52] Bussi G, Donadio D, Parrinello M (2007) Canonical sampling through velocity rescaling. J Chem Phys 126: 014101. 17212484 10.1063/1.2408420

[53] Parrinello M, Rahman A (1981) Polymorphic transitions in single crystals: a new molecular dynamics method. J Appl Phys 52: 7182–7190.

[54] Lyubartsev A, Martsinovski A, Shevkunov S, Vorontsov-Velyaminov P (1992) New approach to Monte Carlo calculation of the free energy: Method of expanded ensembles. J Chem Phys 96: 1776–1783.

[55] Marinari E, Parisi G (1992) Simulated tempering: a new Monte Carlo scheme. Europhys Lett 19: 451–458.

[56] Frishman D, Argos P (1995) Knowledge-based protein secondary structure assignment. Proteins 23: 566–579. 8749853 10.1002/prot.340230412

[57] de Hoon MJ, Imoto S, Nolan J, Miyano S (2004) Open source clustering software. Bioinformatics 20: 1453–1454. 14871861 10.1093/bioinformatics/bth078

[58] Miller RG (1974) The jackknife – a review. Biometrika 61: 1–15.

[59] Delano WL (2011) The PyMOL Molecular Graphics System, Version 1.4.1. Schrödinger, LLC.

