# Supplementary figures and images for: Binding of Two Intrinsically Disordered Peptides to a Multi-Specific Protein: A Combined Monte Carlo and Molecular Dynamics Study
Source: PLoS Comput Biol. 2012 Sep 13;8(9):e1002682. doi: 10.1371/journal.pcbi.1002682 (PMC3441455; doi:10.1371/journal.pcbi.1002682)

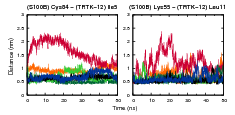

Supplement: Figure S2 — Time evolution of residue-residue distances in MD trajectories of the S100B-TRTK-12 complex. Distances are calculated between the sidechain center-of-mass points of the involved residues, Lys55 and Cys84 for S100B and Ile5 and Leu11 for TRTK-12. (EPS) [file pcbi.1002682.s002.tif]

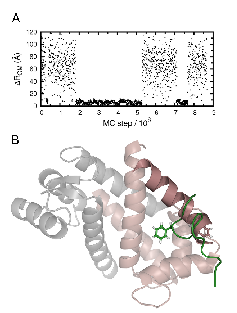

Supplement: Figure S3 — MC protein-peptide binding simulation of S100B and the N terminal regulatory domain of NDR kinase. (A) MC evolution of (see Equation 1 in main text) showing two independent binding/unbinding events, and (B) the minimum-energy conformation found during the trajectory where the simulated peptide is shown in green and the experimental protein-peptide structure is shown in gray and pink (PDB ID 1PSB) [45]. The NDR peptide sequence used in the simulation is KETEFLRLKRTRLGLE. (EPS) [file pcbi.1002682.s003.tif]
